# Anticoagulation for Patients with Venous Thromboembolism: When is Extended Treatment Required?

**DOI:** 10.1055/s-0040-1721735

**Published:** 2020-12-23

**Authors:** Jeffrey I. Weitz, Paolo Prandoni, Peter Verhamme

**Affiliations:** 1 Thrombosis and Atherosclerosis Research Institute and McMaster University, Hamilton, Ontario, Canada; 2 Department of Cardiac, Thoracic and Vascular Sciences, University of Padova, Padua, Italy; 3 Vascular Medicine and Haemostasis, Department of Cardiovascular Medicine, University Hospitals Leuven, Leuven, Belgium

**Keywords:** anticoagulants, recurrence, risk assessment, risk factors, venous thromboembolism

## Abstract

The need for extended venous thromboembolism (VTE) treatment beyond 3 to 6 months is usually determined by balancing the risk of recurrence if treatment is stopped against the risk of bleeding from continuing treatment. The risk of recurrence, and in turn the decision to extend, can be determined through the nature of the index event. Patients with VTE provoked by surgery or trauma (major transient risk factors) are recommended to receive 3 months of anticoagulation therapy because their risk of recurrence is low, whereas patients with VTE provoked by a major persistent risk factor, such as cancer, or those considered to have “unprovoked” VTE, are recommended to receive an extended duration of therapy based on an established high risk of recurrence. Nonetheless, recent evidence and new guidance identify that this approach fails to consider patients with risk factors classed as minor transient (e.g., impaired mobility and pregnancy) or minor persistent (e.g., inflammatory bowel disease and congestive heart disease). Indeed, the risk of recurrence with respect to VTE provoked by minor persistent risk factors has been demonstrated to be not dissimilar to that of VTE without identifiable risk factors. This review provides an overview of the available data on the risk of recurrence according to the underlying cause of VTE, a critical evaluation of evidence from clinical studies on the available anticoagulants for extended VTE treatment, models of risk prediction for recurrent VTE and bleeding, and guidance on how to apply the evidence in practice.

## Introduction

Venous thromboembolism (VTE), encompassing deep vein thrombosis (DVT) and acute pulmonary embolism (PE), affects approximately 1 to 2 in 1,000 individuals annually and is the third most common cause of vascular death after myocardial infarction and stroke. 1 Anticoagulation is the cornerstone of VTE treatment and is recommended for at least 3 months in most patients with DVT and/or PE. The goals of anticoagulant therapy are to avoid fatal PE, to prevent recurrence and to reduce the risk of long-term complications such as postthrombotic syndrome, long-standing exertional dyspnoea, and chronic thromboembolic pulmonary hypertension.

After a 3-month course of anticoagulant therapy, the need for extended treatment is usually determined by balancing the risk of recurrence if treatment is stopped against the risk of bleeding if treatment is continued. 2 However, assessment of the risks of recurrence and bleeding is often a challenging and dynamic process requiring periodic reevaluation of the risks and benefits of extending anticoagulation treatment. Although several models for risk prediction have been developed to aid the decision, all have limitations, and none considers patient preference. Therefore, better methods for identifying patients who require extended anticoagulation therapy are needed.

Previously, the need for extended anticoagulation beyond 3 months was often dichotomized depending on whether the index VTE event was considered “provoked” or “unprovoked.” Traditionally, VTE provoked by a major transient risk factor, such as surgery or trauma, only required a 3-month course of anticoagulation, whereas unprovoked VTE or VTE associated with a major persistent risk factor, such as cancer, required extended therapy beyond 3 months. 2 3 4 Recent evidence and new guidance suggest that this approach is overly simplistic 5 6 because it fails to consider patients with minor transient or minor persistent risk factors. The purpose of this paper is to provide context for the currently available evidence for extended anticoagulation and guidance on the types of patients who may benefit from it.

## Determining the Risk of Recurrence if Anticoagulation Therapy Is Stopped

Determining the risk of recurrence for patients with VTE provoked by major transient or major persistent risk factors is simple. More difficult is determining the risk for recurrence in patients with minor transient or minor persistent risk factors and in those with truly unprovoked VTE. The risk factors within each of these classifications are provided below, and a summary of the 1- and 5-year recurrence rates following discontinuation of anticoagulation therapy for each risk factor category is shown in Table 1 .

**Table 1:** VTE risk factor category descriptions and 1- and 5-year recurrence rates following discontinuation of anticoagulation therapy

Risk factor category	1-year VTE recurrence rate 5 9	5-year VTE recurrence rate 2 57
Major transient	1%	3%
Major persistent	15%	NC a
Minor transient	4–6%	15%
Minor persistent	11%	∼30% b
Unprovoked	8–10%	30%

Abbreviations: NC, not calculable; VTE, venous thromboembolism.

a Unable to calculate due to high mortality.

b Based on the assumption described by Kearon et al in 2010 that the risk of recurrence after stopping anticoagulant therapy conforms to a negatively accelerating curve, and that the cumulative risk of recurrence after 5 years of follow-up is about three times the risk after the first year. 57

### Major Transient Risk Factors

Such risk factors include major surgery and major trauma. 6 7 Examples of major transient risk factors include surgery for hip fracture, hip or knee arthroplasty, and major general surgery, which is often defined as that performed under general anesthesia and lasting for ≥30 minutes. 6 7 8 Major trauma is usually defined as any injury that has the potential to lead to prolonged disability or death. The trauma can be classified as blunt or penetrating in origin and by the area of the body involved such as polytrauma; head injury; or trauma to the chest, abdomen, or extremities. Regardless of the type of surgery or trauma, if the patient has recovered and the risk factor has resolved, the rate of recurrence if anticoagulant treatment is stopped after 3 months is estimated to be approximately 1% at 1 year and 3% at 5 years. With this low risk of recurrence, it is appropriate to stop treatment after 3 months, provided that the risk factor is no longer present. 2 9

### Major Persistent Risk Factors

Examples of major persistent risk factors include cancer and conditions such as antiphospholipid syndrome. Cancer is the most common major persistent risk factor. If anticoagulation is stopped after 3 months in patients with VTE in the setting of metastatic cancer, the annualized risk of recurrence is approximately 15% per year, with an unknown risk at 5 years because of the high mortality rate among such patients. 2 The risk of recurrence with antiphospholipid syndrome varies but is highest in patients with triple positivity (lupus anticoagulant plus immunoglobulin G antibodies against anticardiolipin and β-2 glycoprotein 1). 10 11

### Minor Persistent Risk Factors

Examples of minor persistent risk factors, including inflammatory bowel disease, congestive heart disease, obesity, and their associated risks of VTE recurrence are shown in Table 2 . 5 However, the latest evidence suggests that the risk of recurrence after stopping anticoagulation in these patients is high (a 1-year recurrence rate of 11%) and could be similar to that of unprovoked VTE, especially when the risk factors are truly persistent. 5 Indeed (it is likely that), many patients classified as having unprovoked VTE may, in fact, have an unidentified minor persistent risk factor such as a family history of VTE or thrombophilia.

**Table 2:** Risks of recurrent VTE for minor persistent and minor transient risk factors

Risk factor	Reported risk of recurrent VTE HR unless otherwise stated (CI)	Patient characteristics
**Minor persistent risk factor**		
Renal impairment 58 59	5.32 (1.49–18.95)	Patients with renal impairment (eGFR <60 mL/min/1.73 m ^2^ ) versus those with normal renal function (eGFR ≥90 mL/min/1.73 m ^2^ ) after a previous episode of VTE
	2.84 (1.13–7.11)	Patients with renal impairment (eGFR 60–89 mL/min/1.73 m ^2^ ) versus those with normal renal function after a previous episode of VTE
	1.61 (0.67–3.90)	Patients with a first lifetime VTE with chronic kidney disease versus those without chronic kidney disease at baseline (physician's diagnosis and creatinine level >175 μmol/L [2 mg/dL] for ≥3 months, or nephrotic syndrome)
IBD 58 60	2.5 (1.4–4.2)	Patients with a history of VTE after IBD diagnosis versus those without IBD who had an unprovoked VTE. IBD diagnosis based on clinical, endoscopic, histological, and radiological criteria according to the European Crohn's and Colitis Organization guidelines
	2.37 (1.12–5.01)	Patients with a first lifetime VTE with IBD versus those without IBD at baseline
Lower limb paralysis with extremity paresis 58	1.92 (1.33–2.77)	Patients with a first lifetime VTE with extremity paresis versus those without extremity paresis at baseline
Thrombophilia 61 62	1.9 (1.0–3.9)	Patients with prior VTE and deficiency of antithrombin versus those with no known defect
	RR = 1.5 (1.1–1.9)	Patients with prior VTE with or without heterozygous factor-V Leiden mutation
	RR = 1.4 (1.0–1.8)	Patients with prior VTE with or without heterozygous prothrombin mutation
	1.4 (0.9–2.2)	Patients with prior VTE and deficiency of protein S or protein C versus those with no known defect.
CHD 58	1.43 (1.04–1.97)	CHF or other heart disease (congenital heart disease, cardiomyopathy, ischemic heart disease, or valvular heart disease). Patients with a first lifetime VTE with CHD versus those without at baseline
Family history 63	1.92 (1.44–2.58)	Patients with unprovoked VTE and both parents with history of VTE versus those without
	1.30 (1.14–1.49)	Patients with unprovoked VTE and a sibling with history of VTE versus those without
	1.20 (1.10–1.32)	Patients with unprovoked VTE and one parent with history of VTE versus those without
Obesity 58 64	1.6 (1.0–2.4)	Patients with a first unprovoked VTE with a BMI ≥30 kg/m ^2^ versus those with a BMI <25 kg/m ^2^
	1.21 (0.92–1.60)	Patients with a first lifetime VTE with a BMI ≥30 kg/m ^2^ versus those with a BMI ≥20 to ≤25 kg/m ^2^ at baseline
**Minor transient risk factor**		
Oral estrogen therapy 27 65 a	6.4 (1.5–27.3)	Average of 79-month follow-up of postmenopausal women stratified into users versus nonusers of oral estrogen-based hormone replacement therapy who had ceased anticoagulation treatment after a first confirmed VTE
	RR = 3.5 (2.9–4.3)	Healthy women receiving combined oral contraceptives compared with nonusers
Lower limb injury with impaired mobility 66	OR = 4.5 (1.5–14.0)	Patients with a lower-leg cast within 3 months of a recurrent event versus patients with a cast during a random 3-month follow-up period without a recurrent event
Pregnancy 67	RR: 3.5 (1.6–7.8)	Pregnant versus nonpregnant period in women who had experienced at least one pregnancy following a venous thromboembolic event
Immobilization 68	RR = 2.9 (1.2–7.5)	Immobilized versus ambulant patients with a first episode of VTE receiving a minimum of 3 months of oral anticoagulation therapy
Minor surgery 12	4.6 (1.5–14.2)	Minor surgery versus no surgery in patients with a history of VTE up to 1 month after surgery
Puerperium 34	RR = 1.4 (0.6–3.4)	Puerperium versus pregnant period in women who had experienced at least one pregnancy following a venous thromboembolic event
Travel >8 hours	Not reported	

Abbreviations: BMI, body mass index; CHD, congestive heart disease; CHF, congestive heart failure; CI, confidence interval; eGFR, estimated glomerular filtration rate; HR, hazard ratio; IBD, inflammatory bowel disease; OR, odds ratio; RR, relative risk; VTE, venous thromboembolism.

a Women receiving estrogen replacement therapy after menopause require a higher estrogen potency and have a different baseline level of VTE risk compared with younger women taking estrogen-containing contraceptives. 32

### Minor Transient Risk Factors

Examples of minor transient risk factors, including immobilization, use of oral estrogen therapy, pregnancy, lower limb injury with impaired mobility, and minor surgery, and their respective risks of VTE recurrence are shown in Table 2 . 5 12 The risk of recurrence if anticoagulation therapy is stopped after 3 months in patients with minor transient risk factors varies depending on the risk factor, but is estimated to be approximately 5% at 1 year and 15% at 5 years. Many patients present with multiple minor risk factors (e.g., family history of VTE and long-haul travel), which further complicates the assessment of recurrence risk.

## Direct Oral Anticoagulants Are Effective and Safe for Extended Treatment of VTE

VTE treatment has traditionally been divided into three phases: initial (first 5–10 days), long-term (up to 3 months), and extended (beyond 3 months). Direct oral anticoagulants (DOACs) are replacing warfarin for the initial, long-term, and extended treatment of VTE, with guidelines now giving preference to them over vitamin-K antagonists (VKAs). Evidence of the efficacy and safety of DOACs for extended VTE treatment comes from studies comparing them with placebo, acetylsalicylic acid (ASA), or warfarin. Studies with apixaban or rivaroxaban for extended VTE treatment indicate that their efficacy is maintained even if the dose of the drug is lowered. Lower dose regimens are likely to enhance the benefit–risk profile of DOACs, even further than full-dose regimens. Phase-III trial results (including a pooled analysis of two studies), the findings of a phase-III study post hoc analysis, and real-world evidence evaluating the DOACs for extended VTE treatment are summarized in Table 3 and are briefly described below. 13 14 15 16 17 18 19 20 21 22 23 24

**Table 3:** Overview of DOAC studies of extended VTE treatment and secondary prevention

	AMPLIFY-EXT 16 *n* = 2,486	RE-MEDY 13 *n* = 2,866	RE-SONATE 13 *n* = 1,353	EINSTEIN EXT 14 *n* = 1,197	EINSTEIN CHOICE 15 *n* = 3,396	Hokusai-VTE post hoc analysis 17 *n* = 7,227 a
DOAC (dosing regimen)	Apixaban (5-mg bid or 2.5-mg bid)	Dabigatran (150-mg bid)	Rivaroxaban (20-mg od)	Rivaroxaban (20-mg od or 10-mg od)	Edoxaban (60-mg od) b
Comparator	Placebo	Warfarin	Placebo	ASA 100-mg od	Warfarin
Duration of prior anticoagulation (mo)	6–12	3–12	6–18	6–12	None
Study duration (mo)	12	6–36	6–18	6 or 12	12	3–12
Primary efficacy endpoint (vs. comparator)	Recurrent VTE or all-cause mortality (2.5-mg bid): 3.8 vs. 11.6%; *p * < 0.001 Recurrent VTE or all-cause mortality (5-mg bid): 4.2 vs. 11.6%; *p * < 0.001	Recurrent or fatal VTE: 1.8 vs. 1.3%; *p* = 0.01 for noninferiority	Recurrent or fatal VTE: 0.4 vs. 5.6%; *p * < 0.001	Recurrent VTE: 1.3 vs. 7.1%; *p * < 0.001	Symptomatic recurrent fatal or nonfatal VTE (20-mg od):1.5 vs. 4.4%; *p * < 0.001 Symptomatic recurrent fatal or nonfatal VTE (10-mg od): 1.2 vs. 4.4%; *p * < 0.001	Symptomatic recurrent VTE: 0.3 vs. 0.4%; *p* = NR
Primary safety endpoint (vs. comparator)	Major bleeding (2.5-mg bid): 0.2 vs. 0.5%; *p* = NR Major bleeding (5-mg bid): 0.1 vs. 0.5%; *p* = NR	Major bleeding: 0.9 vs. 1.8%; *p* = 0.06	Major bleeding: 0.3 vs. 0%; *p* = 1.0	Major bleeding: 0.7 vs. 0%; *p* = 0.11	Major bleeding (20-mg od): 0.5 vs. 0.3%; *p* = 0.32 Major bleeding (10-mg od): 0.4 vs. 0.3%; *p* = 0.50	Major bleeding: 0.3 vs. 0.7%; *p* = NR
Risk factors at baseline (%)						
Active cancer	1.7	4.2	0.2	4.5	2.6	NR
Unprovoked VTE	91.7	NR	73.7	41.3	67.3

Abbreviations: ASA, acetylsalicylic acid; bid, twice daily; CrCl, creatinine clearance; DOAC, direct oral anticoagulant; NR, not reported; od, once daily; VTE, venous thromboembolism.

a The Hokusai-VTE study randomized 8,292 patients, but only 7,227 were eligible for the post hoc extended treatment analysis.

b A dose of edoxaban 30-mg od was used in patients with CrCl of 30–50 mL/min, body weight ≤60 kg or in patients who were receiving concomitant treatment with potent P-glycoprotein inhibitors.

### Apixaban: AMPLIFY-EXT

In AMPLIFY-EXT, two doses of apixaban (5-mg twice daily [bid] and 2.5-mg bid) were compared with placebo in patients who had completed 6 to 12 months of anticoagulation therapy. Both doses were associated with a reduced risk of the primary outcome of recurrent VTE or death from any cause compared with placebo (relative risk [RR] = 0.36, 95% confidence interval [CI]: 0.25–0.53; and RR = 0.33, 95% CI: 0.22–0.48, respectively; *p* < 0.001), without an increase in the incidence of major bleeding (0.1, 0.2, and 0.5%, respectively). The RRs of the composite endpoint of major or clinically relevant nonmajor (CRNM) bleeding with apixaban 5-mg bid and apixaban 2.5-mg bid were not significantly increased compared with placebo (RR = 1.62, 95% CI: 0.96–2.73 and RR = 1.20, 95% CI: 0.69–2.10, respectively). 16 The study was powered to assess the superiority of apixaban versus placebo for the primary efficacy outcome of recurrent VTE or all-cause death; it was not powered to compare the two doses of apixaban or to assess differences in the safety outcome between treatment arms. Therefore, definitive inferences about the efficacy and safety of the two apixaban doses are not possible. 16

### Dabigatran: RE-MEDY and RE-SONATE

In two double-blind randomized trials, dabigatran 150-mg bid was compared with either warfarin (active control) or placebo in patients who had completed ≥3 months of anticoagulation therapy. In RE-SONATE, dabigatran significantly reduced the incidence of recurrent or fatal VTE or unexplained death versus placebo (hazard ratio [HR] = 0.08, 95% CI: 0.02–0.25; *p* < 0.001), and in RE-MEDY, dabigatran was noninferior to warfarin (HR = 1.44, 95% CI: 0.78–2.64; *p* = 0.01). The incidence of major bleeding was not significantly different with dabigatran versus warfarin or placebo (0.9 vs. 1.8 and 0.3 vs. 0%, respectively). However, the incidence of the composite of major or CRNM bleeding with dabigatran was significantly lower than that with warfarin (HR = 0.54, 95% CI: 0.41–0.71; *p* < 0.001), but significantly higher than that with placebo (HR = 2.92, 95% CI: 1.52–5.60; *p* = 0.001). 13

### Edoxaban: Hokusai-VTE

There has been no dedicated extension study with edoxaban. However, a post hoc analysis of the Hokusai-VTE phase-III trial was conducted in patients who were treated with edoxaban or warfarin for at least 3 months and in whom treatment was continued for up to 12 months. The cumulative incidence of symptomatic nonfatal and fatal VTE between 3 and 12 months was similar between the treatment groups, with incidences of recurrent VTE of 0.3% in the edoxaban-treated group and 0.4% in the warfarin-treated group. The incidence of major bleeding was lower with edoxaban than with warfarin (0.3 vs. 0.7%; HR = 0.45, 95% CI: 0.22–0.92). 25

### Rivaroxaban: EINSTEIN EXT and EINSTEIN CHOICE

EINSTEIN EXT compared rivaroxaban 20-mg once daily (od) with placebo for 6 or 12 months in patients who had completed 6 to 12 months of prior anticoagulation therapy. The incidence of recurrent VTE was significantly lower with rivaroxaban than with placebo (HR = 0.18, 95% CI: 0.09–0.39; *p* < 0.001), whereas the incidence of major bleeding was not significantly increased (0.7 and 0% with rivaroxaban and placebo, respectively; *p* = 0.11), but there was more CRNM bleeding with rivaroxaban than with placebo (5.4 and 1.2%, respectively). 14

EINSTEIN CHOICE compared the efficacy and safety of the treatment with two doses of rivaroxaban (20-mg od and 10-mg od, respectively) versus ASA (100-mg od) for secondary prevention in patients who had completed 6 to 12 months of anticoagulation. Both the high and the low doses of rivaroxaban regimen significantly reduced the incidence of recurrent symptomatic fatal or nonfatal VTE compared with ASA (HR = 0.34, 95% CI: 0.20–0.59 and HR = 0.26, 95% CI: 0.14–0.47, respectively; *p* < 0.001 for both comparisons). The incidence of major bleeding was similar in all groups (0.5% with rivaroxaban 20-mg od, 0.4% with rivaroxaban 10-mg od, and 0.3% with ASA). 15 This study was powered to assess the superiority of rivaroxaban versus ASA for the primary efficacy outcome; it was not powered to compare the efficacy and safety of the two rivaroxaban doses, or to detect differences in the safety outcome between treatment arms. Therefore, definitive inferences about the efficacy and safety of the two rivaroxaban doses are not possible. 15

### A Pooled Analysis of EINSTEIN EXT and EINSTEIN CHOICE Showed That the Risk of Recurrence with VTE Provoked by Minor Persistent Risk Factors is Like That With Unprovoked VTE

A pooled analysis of EINSTEIN EXT and EINSTEIN CHOICE provides a novel perspective on the risk of recurrence in patients with VTE provoked by minor persistent or minor transient risk factors. The analysis confirmed that the risk of recurrence after stopping anticoagulation among patients with unprovoked VTE was high, whereas the risk was low in those with VTE provoked by a major transient risk factor. In patients with unprovoked VTE, recurrence occurred in 1.6% (19/1,173) of patients who received rivaroxaban, 5.5% (26/468) of patients who received ASA, and 8.2% (20/243) of patients who received placebo. The corresponding cumulative 1-year incidences for recurrent VTE were 2.0, 5.9, and 10.0%, respectively. 5

In patients with VTE provoked by major transient risk factors, there were no recurrences in any treatment group. In contrast, the rates of recurrence in patients with VTE provoked by minor persistent or minor transient risk factors were not significantly lower than in patients with unprovoked VTE (HR = 0.81, 95% CI: 0.56–1.16 and HR = 0.68, 95% CI: 0.32–1.30, respectively). Minor persistent risk factors included renal impairment, family history of VTE, and lower extremity paralysis or paresis; minor transient risk factors included travel >8 hours in duration, use of estrogen therapy and pregnancy, and leg injury with impaired mobility. These findings suggest that some patients with VTE provoked by minor persistent or minor transient risk factors should also be considered for extended anticoagulation (dependent on their risk of bleeding). 5

### Real-World Studies

Results from randomized controlled trials (RCTs) may not translate fully into clinical practice owing to differences in medication environments (controlled vs. clinical practice), patient populations (homogeneous vs. heterogeneous), and adherence to medication. Real-world evidence can help reinforce results from phase-III studies. The results from the pooled XALIA studies (a large prospective real-world dataset on the use of rivaroxaban for the treatment of VTE) and the SWIVTER, PREFER in VTE, GARFIELD-VTE, REMOTEV, Dresden non-VKA oral anticoagulant and RIETE registries have reported reductions in the incidence of recurrent VTE and major bleeding associated with DOACs compared with VKAs similar to those found in the RCTs. These real-world studies provide additional support for the efficacy and safety of anticoagulation in patients treated in routine practice to help inform clinical decision-making on extended anticoagulation. 18 19 20 21 22 23 24

## Current Guideline Recommendations: Where Is the Evidence?

Guidelines recommend a minimum of a 3-month course of anticoagulation therapy for all patients with VTE. 6 26 The 2019 update of the European Society of Cardiology (ESC) guidelines for acute pulmonary embolism (PE) was the first to consider the latest evidence regarding duration of therapy and the broader risk factor definitions. 6 These guidelines now state that indefinite anticoagulation should be considered for patients with a first episode of PE that is unprovoked or associated with a persistent risk factor. DOACs are preferred over VKAs except in patients with antiphospholipid syndrome. Extended anticoagulation is also to be considered after a first episode of PE associated with a minor risk factor but not routinely in those episodes associated with pregnancy or estrogen therapy. Indefinite anticoagulant therapy is recommended for patients with a recurrent VTE event not related to a known risk factor. 2 6 The guidelines further state that for extended oral anticoagulation in patients with PE without cancer, apixaban and rivaroxaban should be considered at a reduced dose after the first 6 months of therapy. 26

Estrogen use is considered a minor transient risk factor for VTE, 7 27 28 and the risk of recurrence after an initial estrogen-associated VTE is generally low provided that estrogen therapy is stopped. 29 30 The risk of an initial VTE associated with estrogen replacement therapy or estrogen-containing contraceptives is also generally low and depends on several factors, including the type of preparation, route of administration, patient age, and the duration of treatment. 31 32 Oral estrogen use is estimated to increase the risk of VTE two to six fold compared with nonuse, with an absolute risk of approximately 1 to 3 cases per 10,000 woman-years. However, the risk of VTE is highest in the first 6 to 12 months after initiating estrogen therapy, and decreases over time thereafter. 32 The risk increasing with estrogen use is still within a range consistent with the definition of minor transient risk factors according to the guidelines. 2 6 7 32 In young female patients with PE associated with estrogen-containing oral contraceptives, particularly in cases where estrogen therapy was initiated within 3 months prior to PE, the 2019 ESC guidelines on treatment of acute PE recommend using an alternative form of contraception and discontinuing anticoagulation after 3 months. 6 In patients who do not meet these criteria, the guidelines suggest quantifying the risk of VTE recurrence and managing long-term anticoagulation therapy as in patients with acute PE with no identifiable risk factors. 6

Pregnancy has also been identified as a minor transient risk factor for VTE. 2 6 7 The risk of VTE recurrence is relatively low after an initial pregnancy-associated VTE but increases again in subsequent pregnancies and puerperium. Direct evidence on the optimal duration of anticoagulation after pregnancy-associated VTE is not yet available. 6 33 34 35 Therefore, extended anticoagulation is not routinely recommended in this setting. The 2019 ESC guidelines on acute PE recommend anticoagulation with low molecular weight heparin (LMWH) throughout pregnancy and for at least 6 weeks postpartum after an acute PE, and suggest that the patient should be advised of the need for anticoagulation therapy during subsequent pregnancies. 6 LMWH therapy is the preferred anticoagulant in these patients because warfarin and DOACs are contraindicated or not recommended during pregnancy, and DOACs but not warfarin are contraindicated or not recommended during lactation. 2 6 36 37 38 39 40 The 2012 CHEST guidelines provide guidance on the duration of anticoagulation therapy for VTE associated with pregnancy and recommend that anticoagulants to be continued for at least 6 weeks postpartum (for a minimum duration of therapy of 3 months) rather than shorter durations of treatment. 41

Guidance from the International Society on Thrombosis and Haemostasis (ISTH) states that edoxaban and rivaroxaban are currently the only DOACs that have been compared with LMWH in RCTs in cancer populations. 42 The ISTH guidance also suggests that specific DOACs, which have been evaluated in RCTs, should be used in patients with cancer and an acute diagnosis of VTE, a low-risk of bleeding, and no drug–drug interactions with current systemic therapy (LMWH is an acceptable alternative). Patients with acute VTE and a high risk of bleeding are recommended to receive LMWH, with edoxaban or rivaroxaban as suitable alternatives. Guidance on the length of anticoagulation therapy in this setting is not provided by the ISTH. 42 The American Society of Clinical Oncology guidelines and the International Initiative on Thrombosis and Cancer now recommend the use of LMWH, edoxaban, or rivaroxaban for a least 6 months to reduce the risk of recurrence in patients with cancer and established VTE. 3 4 Anticoagulation beyond 6 months can be offered to selected patients with active cancer, those with metastatic disease, or those undergoing chemotherapy or treatment with other anticancer agents. Anticoagulation needs should be assessed on an intermittent basis to ensure a favorable benefit–risk profile. 3 4

It is expected that recommendations in future updates to older guidelines on the duration of secondary prevention for VTE will also be amended in response to the latest evidence from the DOAC trials. For example, the recently published CARAVAGGIO study showed that apixaban 5-mg bid had noninferior efficacy to dalteparin for the treatment of cancer-associated VTE (HR = 0.63, 95% CI: 0.37–1.07; *p* < 0.001 for noninferiority) and was not associated with a significant increase in major bleeding (HR = 0.82, 95% CI: 0.40–1.69; *p* = 0.60). 43 The API-CT study is investigating full-dose versus reduced-dose apixaban for the long-term treatment of VTE in cancer. 44 Guidelines also recommend the use of VKAs for extended anticoagulation in specific patient populations, such as patients with antiphospholipid syndrome. When VKAs are proposed as the treatment choice, they should be dose adjusted to achieve an international normalized ratio of 2.0 to 3.0. 4 6 26

## Predicting the Risk of Bleeding and Recurrent Venous Thromboembolism

### Bleeding Risk Factors

To date, the following have been identified as risk factors for bleeding: advanced age, previous bleeding events or anemia, active cancer, previous stroke (hemorrhagic or ischemic), chronic renal or hepatic disease, concomitant antiplatelet therapy or use of nonsteroidal anti-inflammatory drugs, other serious acute or chronic illness, and poor anticoagulation control. 45 Bleeding prevention strategies can also be considered for modifiable risk factors (e.g., treatment for hypertension, minimizing duration and intensity of simultaneous nonsteroidal anti-inflammatory drugs and antiplatelet therapy, and moderating alcohol intake). 46

### Bleeding Risk Tools

A predictive model of note is VTE-BLEED, a tool used to predict major bleeding during chronic anticoagulation for VTE. 47 VTE-BLEED is based on following six variables: (1) active cancer, (2) male sex with uncontrolled arterial hypertension, (3) anemia, (4) history of bleeding, (5) age ≥60 years, and (6) renal dysfunction. 47 The main benefit of VTE-BLEED is that it can differentiate between patients with VTE with a higher or lower risk of bleeding during long-term anticoagulation (≥90 days). 47 Although VTE-BLEED was validated in international and “real-world” cohorts, it was evaluated in an unselected patient cohort in Japan. 48 Due to potential population-specific differences in the baseline risk of VTE and bleeding, however, the results of the Japanese study cannot be generalized to other populations.

HAS-BLED (Hypertension, Abnormal renal/liver function, Stroke, Bleeding history or predisposition, Labile international normalized ratio, Elderly, Drugs/alcohol concomitantly) is another well-known bleeding risk score that was originally developed to estimate the risk of major bleeding in patients with atrial fibrillation. Evaluation of its predictive value for major bleeding risk has more recently been demonstrated in the first 6 months of anticoagulation therapy in patients with VTE but further adaptations and validation may be warranted. 49 50

### Venous Thromboembolism Risk Factors

Risk factors and clinical presentation can provide guidance on extended anticoagulation for many patients with VTE but not all. Additional factors to consider when there is the choice of extending anticoagulation are D-dimer levels, clinical presentation (extensive proximal DVT vs. distal DVT, segmental or subsegmental PE vs. massive or submassive PE, and high-risk or intermediate high-risk PE), presence of postthrombotic syndrome or post-PE symptoms, patient preference, familial history of VTE, and thrombophilia testing (to uncover persistent risk factors). There are also some strategies that can be employed to help clinicians. Two such strategies are formal risk assessment models for recurrent VTE risk and risk of bleeding if anticoagulation is continued.

### Venous Thromboembolism Risk Tools

The Vienna Prediction Model is based on the following three key parameters: (1) patient sex, (2) site of VTE, and (3) D-dimer level. This model allows prediction at 3 weeks and 3, 9, 15, and 24 months after stopping anticoagulation ( Table 4 ). 26 51 52 However, it is a complex tool and difficult to apply at the bedside without the use of a digital application.

**Table 4:** Overview of clinical prediction tools assessing risk of recurrent VTE 26 55

Score	Vienna prediction model	DASH score	Men Continue and HERDOO2
Parameters	D-dimer level at 3 weeks and 3, 9, 15, and 24 months after stopping anticoagulation Male sex VTE location (distal DVT, proximal DVT, PE)	Abnormal D-dimer 3–5 weeks after stopping anticoagulation Male sex Age <50 years VTE not associated with estrogen-progestogen therapy in women	Abnormal D-dimer (≥250 μg/L) before stopping anticoagulation Postthrombotic symptoms (hyperpigmentation, edema, and redness) Age ≥65 years BMI ≥30 kg/m ^2^
Validation study	Yes	Yes	Yes
Recurrence risk	Different nomograms to calculate risk of VTE recurrence at different times	Patients with low score (≤1) have an annual recurrence rate of 3.1%	Is applicable to women only. Women with low score (≤1) have an annual recurrence rate of 1.3–1.6%

Abbreviations: BMI, body mass index, DVT, deep vein thrombosis; HERDOO2, Hyperpigmentation, Edema, or Redness in either leg; D-dimer level ≥ 250 μg/L; Obesity with body mass index ≥ 30; or Older age, ≥ 65 years; PE, pulmonary embolism; VTE, venous thromboembolism.

The DASH (D-dimer, Age, Sex, Hormonal therapy) score predicts which patients are at low risk of VTE recurrence and, therefore, can stop anticoagulation after an appropriate 3- to 6-month period of treatment. The score is based on D-dimer levels, age, sex, and hormone treatment, and patients who score ≥2 should be considered at high risk of recurrent VTE and, therefore, eligible for extended VTE treatment. The DASH score has been externally validated and its utility in younger patients was confirmed; however, the risk of recurrence in patients >65 years of age is >5%, even in patients with low DASH scores ( Table 4 ). It should be noted that the overwhelming majority of anticoagulation in this scoring system was with VKAs, and there is limited evidence of the use of the DASH score with other drug classes. 53

The Men Continue and Hyperpigmentation, Edema, or Redness in either leg; D-dimer level ≥ 250 μg/L; Obesity with body mass index ≥ 30; or Older age, ≥ 65 years (HERDOO2) score operates on the principle that men with unprovoked VTE should continue anticoagulation treatment, whereas women with unprovoked VTE and a score ≥2 should continue. Risk factors considered are D-dimer levels ≥250 µg/mL on anticoagulant therapy, age ≥65 years, body mass index ≥30 kg/m ^2^ , and features of postthrombotic syndrome such as hyperpigmentation, edema, and redness ( Table 4 ). 54 55 This score has only been applied to patients with unprovoked VTE and women with estrogen-associated VTE.

It should be noted that while all the VTE risk scores use D-dimer level as a variable, Men Continue and HERDOO2 measure it while patients are on anticoagulation, whereas the Vienna model and DASH score assess D-dimer levels after stopping treatment. 51 53 54 Positive D-dimer measurements are less useful for predicting VTE recurrence in men than in women because even men with a normal D-dimer level after stopping anticoagulant therapy have a sufficiently high risk of recurrence to warrant extended therapy. 2 Although these risk scores are useful, they should be used with caution until further external, robust validation is performed on new data. Finally, data from the DULCIS study support a strategy where anticoagulation can be stopped based on serial D-dimer measurements showing persistently negative results, as these patients appear to be at low risk of VTE recurrence. 56

## Who Should Receive Extended Venous Thromboembolism Treatment?

**Fig. 1:**
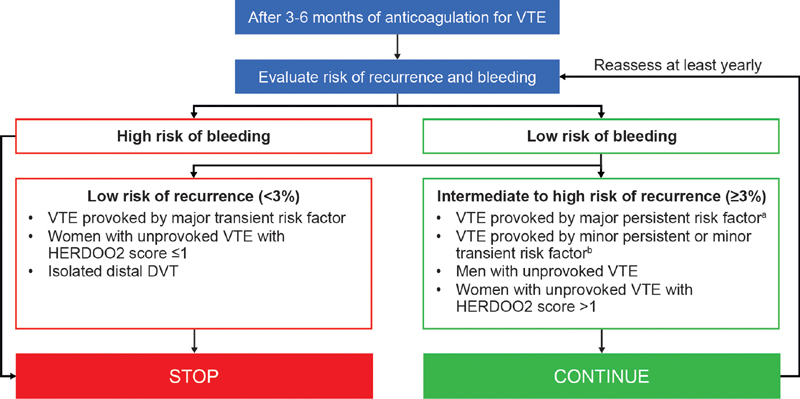
Decision tree for continued anticoagulation in patients with VTE. 2 4 5 6 26 54 ^a^ Major persistent conditions include active cancer and antiphospholipid antibody syndrome. ^b^ Minor persistent defined as inflammatory bowel disease, lower extremity paralysis or paresis, congestive heart failure, BMI >30 kg/m ^2^ , creatinine clearance <50 mL/min, family history of VTE, hereditary thrombophilia and acquired thrombophilia. Minor transient defined as immobilization, travel >8 hours and leg injury with impaired mobility. BMI, body mass index; DVT, deep vein thrombosis; HERDOO2, Hyperpigmentation, Edema, or Redness in either leg; D-dimer level ≥ 250 μg/L; Obesity with body mass index ≥ 30; or Older age, ≥ 65 years; VTE, venous thromboembolism.

Based on the latest guideline recommendations for patients with VTE and the latest evidence in the field, a stepwise approach should be considered, as outlined in Fig. 1 . The decision to extend anticoagulation therapy depends on the associated benefits versus risks, which may change with time. Furthermore, patients receiving extended anticoagulation therapy should be reassessed at least on a yearly basis to determine their risk of VTE recurrence and bleeding. 2 6

The benefit–risk profile of extending anticoagulation in the era of “low-dose” DOACs is different than it was in the VKA era, because of the improved safety profile and convenience associated with DOACs. When not opting for continued secondary prevention, thromboprophylaxis in high-risk situations, clinical vigilance, and timely diagnosis are valid alternatives for many patients (“treatment” does not stop when pharmacological agents are discontinued).

## Conclusion

Guidance for the extension of anticoagulation beyond 3 months has traditionally been clear for patients with VTE provoked by a major transient risk factor (e.g., surgery) or a major persistent risk factor (e.g., cancer). However, debate has existed over whether anticoagulation should be extended in the case of minor persistent or minor transient risk factors. New evidence in the latter settings from clinical studies involving DOACs and updates to international guidelines, along with risk prediction tools, can help guide and inform clinical decisions on extending anticoagulation treatment in patients with such risk factors. Additional guidance on anticoagulation duration in special circumstances such as pregnancy and contact sports would also be of value in the future.
